# Using hyperspectral imaging and machine learning to identify food-contaminated compostable and recyclable plastics

**DOI:** 10.14324/111.444/ucloe.3237

**Published:** 2025-04-02

**Authors:** Nutcha Taneepanichskul, Helen C. Hailes, Mark Miodownik

**Affiliations:** 1 Mechanical Engineering Department, University College London, London, UK; 2 Chemistry Department, University College London, London, UK

**Keywords:** food-contaminated plastics, hyperspectral imaging (HSI), recycling, composting, machine learning, automatic sorting

## Abstract

With the increasing public legislation aimed at reducing plastic pollution, compostable plastics have emerged as an alternative to conventional plastics for some food packaging and food service items. However, the true value of compostable plastics can only be realised if they do not enter the environment as contaminants but instead are processed along with food waste using industrial composting facilities. Distinguishing compostable plastics from other plastics in this waste stream is an outstanding problem. Currently, near-infrared technology is widely used to identify polymers, but it falls short in distinguishing plastics contaminated with food waste. This study investigates the application of hyperspectral imaging to address this challenge, enhancing the detection and sorting of contaminated compostable plastics. By combining hyperspectral imaging with various machine learning algorithms we show it is possible to accurately identify and classify plastic packaging with food waste contamination, achieving up to 99% accuracy. The study also measures the impact of plastic features such as darkness, size and level of contamination on model performance, with darkness having the most significant impact. The developed machine learning model can detect plastic with higher levels of contamination more accurately compared to our previous study. Implementing hyperspectral imaging in waste management systems can significantly increase composting and recycling rates, and improve the quality of recycled products. This advanced approach supports the circular economy by ensuring that both compostable and recyclable plastics are effectively processed and recycled, minimising environmental impact.

## Introduction

The increasing popularity of compostable and biodegradable plastics underscores the need for efficient sorting technologies to separate and collect them for waste processing. In 2023 they represented 52.1% of the global bioplastic production [1]. In the current UK waste management system, organic waste is managed through two primary methods: in-vessel composting (IVC) and anaerobic digestion (AD). Compostable and biodegradable plastics should be directed to IVC, where they can break down into compost. In contrast, AD is not suitable for compostable plastics, as they can clog the system. However, the systems often fail to detect and separate compostable plastics, especially when contaminated with food waste, leading to their improper disposal in landfills, incinerators or misdirection to AD or recycling plants. This significantly contributes to low recycling and composting rates [2].

Near-infrared (NIR) optical sorting is a widely used technology in recycling facilities for separating different types of plastics [3]. NIR offers significant advantages over RGB-based imaging methods, as it relies on the distinct spectral signatures of various plastic polymers to achieve accurate sorting. In contrast, RGB-based methods often struggle to identify plastics with varying transparency, colour and quality [4]. Combining RGB with NIR imaging can enhance the model’s overall performance [5–7]. However, food waste contamination poses significant challenges to the efficiency and effectiveness of NIR optical sorting due to issues with spectral abortion reflection of NIR frequencies by the food residues on the plastic surfaces [8]. Additionally, the presence of food waste introduces extra spectral signals in the NIR range, creating noise that makes it harder for the system to accurately identify the polymer type.

Hyperspectral imaging (HSI) coupled with machine learning (ML) algorithms offers an advanced solution for sorting plastics, surpassing traditional NIR optical sorting methods. HSI generates a hyperspectral cube, where each pixel contains a continuous spectrum, enabling detailed spectral analysis at each pixel in the image. This capability helps overcome the challenges posed by food-contaminated plastics because uncontaminated pixels can be correctly identified rather than relying on the average signal from the whole sample as with NIR methods.

While numerous studies have explored the application of HSI for identifying various types of plastics, there remains a notable gap in research that specifically addresses the challenge of detecting food-contaminated compostable plastics, which represents a significant issue within the context of current plastic waste management systems.

In 2013 Ulrici et al. used HSI and partial least squares discriminant analysis (PLS-DA) to distinguish polyethylene terephthalate (PET) and polylactic acid (PLA) achieving over 98% accuracy with just six variables on the reduced matrix [9]. Subsequently, Bonifazi used HSI with ML to sort paper, cardboard, plastics and multilayer packaging. A PLS-DA-based model achieved a 0.933 recognition and reliability rate, making HSI a reliable, low-cost solution for identifying impurities and composite materials in plastic waste streams [10]. Taneepanichskul et al. then applied HSI [11] together with PLS-DA to identify and classify compostable plastics [PLA and polybutylene adipate terephthalate (PBAT)], compostable materials (sugarcane and palm leaf derived packaging) and conventional plastics [low-density polyethylene (LDPE), PET and polypropylene (PP)]. PLS-DA achieved a perfect classification (100%) for virgin materials larger than 10 mm × 10 mm [12]. Taneepanichskul et al. also recently studied the impact of packaging properties such as darkness, colour, size and contamination, showing how they all impacted identification. The accuracy of the system decreased when detecting plastics that were dark, thin, small or had high levels of contamination [11].

Currently, no research has been conducted on identifying plastics contaminated with food waste, which constitutes a significant portion of plastic waste in the UK waste management system. In this paper, we present the development of chemometric, and ML algorithms integrated with HSI, demonstrating their effectiveness in identifying compostable and recyclable plastics with varying types and levels of food contamination. This study improves upon our previous works [11,12], which focused solely on virgin plastics and those contaminated with compost from IVC. The enhanced models demonstrate excellent performance, effectively detecting plastics with diverse types and varying levels of food contamination. The overall model accuracy for identifying contaminated plastics has improved from 85% to 97%. Additionally, the study explores the impact of real-world food plastic packaging properties such as size, colour and darkness, on the performance of the system.

The remainder of this manuscript is structured as follows: there is a Materials and methods section which details the simulation of food contamination, the materials used to construct the training, cross-validation and testing datasets, and the process of hyperspectral image acquisition and analysis. Additionally, the performance of the classification models is analysed, and the algorithm for evaluating plastic features, including size, darkness, contamination levels and food contaminant colours is described. In the Results section, the average raw and pre-processed spectra are presented, and the performance of each classification model is calculated and compared. The impact of plastic features (size, darkness, contamination levels and food contaminant colours) on the accuracy of the developed classification model is analysed. In the Discussion and Conclusion sections, the results are compared with findings from our previous studies as well as other related studies to provide valuable insight. Additionally, the use of HSI for increasing the efficiency of detecting food-contaminated compostable plastics in the waste streams of AD, IVC and recycling plants is discussed.

## Materials and methods

To develop the model to identify and classify food-contaminated compostable and recyclable plastic packaging, samples were required for the development of three datasets: a calibration dataset, a cross-validation dataset and a testing dataset. The training dataset is the initial set of data used to train a model [13]. The cross-validation dataset evaluates the model’s predictive performance on new, unseen data, helping to identify issues like overfitting or selection bias and providing insight into the model’s ability to generalise to an independent dataset [14]. The testing dataset offers a final, real-world validation of the model’s effectiveness on completely unseen data [15]. The details of the food contaminants, the plastic samples, the HSI system and the deep learning algorithms are described in the following sections.

### Simulating food contamination

**Figure 1:**
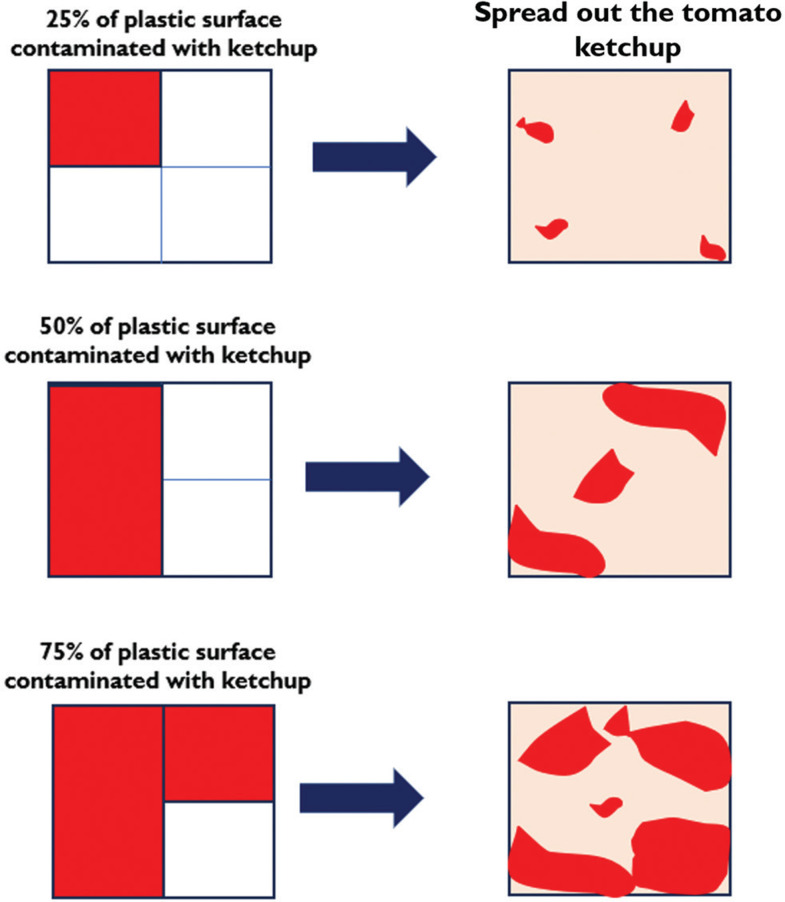
Simulated contamination levels of 25%, 50% and 75% sauces.

The contamination levels in this experimental setup were categorised into three levels: low (25%), medium (50%) and high (75%). Figure 1 illustrates the contamination process, depicting the simulation of 25%, 50% and 75% contamination using tomato ketchup. Each sample was cut into 50 mm × 50 mm pieces with a thickness of 0.4 mm and divided into four equal sections.

Two sauces were used to simulate food contamination: tomato ketchup and mayonnaise. These were chosen due to their ability to be applied repeatably and consistently to the samples. The different compositions help to create training data and cross-validation data. The compositions of these two sauces are shown in Table 1. These condiments are suitable proxies for food contamination because they can represent high water activity foods such as dips and sauces, prepared salads and dairy products; acidic foods such as pickled products, fermented foods and fruit-based sauces; emulsified foods such as salad dressings, processed meats and butter and margarine; and fat-containing foods. Their compositional similarities to a wide range of other food products make them ideal for studying contamination and spoilage patterns across different food categories.

**Table 1.** The ingredients and components of Heinz tomato ketchup and Heinz mayonnaise

Heinz tomato ketchup main ingredient	Component
Tomatoes	Water
	Carbohydrate: including sugars (glucose and fructose) and dietary fibre
	Acid: citric acid and malic acid, contributing to the tartness
	Vitamin: vitamin C, vitamin A (from beta-carotene) and vitamin K
	Minerals: potassium, magnesium and iron
	Antioxidants: lycopene, which gives tomatoes their red colour
Vinegar	Acetic acid
Sugar	Glucose, fructose, sucrose
Salt	Sodium chloride
Olive oil	Monounsaturated fats: predominantly oleic acid
	Antioxidants: polyphenols and vitamin E
	Fat-soluble vitamins: vitamins K and E

Heinz mayonnaise	Component
Oil	Triglycerides
	Fatty acid
Egg yolk	Water
	Proteins
	Fats
	Cholesterol
	Vitamins A, D, E and K
Vinegar	Acetic acid
Water	-
Sugar	Glucose, fructose, sucrose
Starch	-
Salt	Sodium chloride
Mustard	Water, acids, vitamin A and vitamin C

Additionally, their viscosity and texture allow them to adhere well to surfaces, effectively simulating real-life conditions of food residue on plastics. This makes them ideal for testing cleaning and contamination processes. In this study, Heinz tomato ketchup was used for both the training and cross-validation datasets, while Heinz mayonnaise was used for the cross-validation dataset.

To achieve 25% contamination, tomato ketchup or mayonnaise was applied to one section; for 50% contamination, it was applied to two sections; and for 75% contamination, it was applied to three sections. The ketchup and mayonnaise were then spread to ensure they covered the entire plastic surface.

### Sample preparation

The experimental samples encompassed several size and contamination levels, with both conventional and compostable plastics. Within the category of conventional plastics, LDPE, high-density polyethylene (HDPE), PET and PP were represented. The compostable plastic category comprised PLA, PBAT and polyhydroxyalkanoate (PHA).

The materials were allocated into three datasets, namely calibration, cross-validation and testing datasets as mentioned earlier. The training dataset encompassed both pristine plastics and plastics contaminated with a low level of tomato ketchup (25%). This approach allowed the models to learn patterns associated with both clean and contaminated plastics. It also helped the models identify the essential features of plastics from pristine samples while adapting to variations introduced by a specific type of contamination (tomato ketchup). The details of the materials within the training dataset are presented in Table 2.

**Table 2.** List of samples in training dataset

Material	Material condition	Size	Number of replicates per plastic type
LDPE, HDPE, PET, PP, PLA, PBAT and PHA	Pristine plastic	50 mm × 50 mm	35
		40 mm × 40 mm	35
		30 mm × 30 mm	35
		20 mm × 20 mm	35
	Plastics with 25% level of tomato ketchup contamination	50 mm × 50 mm	21

In the cross-validation set, there were three replicates each with 50% and 75% tomato ketchup contamination of the plastic samples and 25%, 50% and 75% mayonnaise contamination of the plastic samples as shown in Table 3. The models were validated using high levels of contamination from tomato ketchup and a new type of contamination (mayonnaise) to assess their robustness. This approach ensured that the models could effectively handle complex scenarios and accurately identify plastic types, even under higher levels of tomato ketchup contamination and new contamination conditions (mayonnaise).

**Table 3.** List of samples in cross-validation dataset

Material	Material condition	Size	Number of replicates per plastic type
LDPE, HDPE, PET, PP, PLA, PBAT and PHA	Plastics with 50% level of tomato ketchup contamination	50 mm × 50 mm	21
	Plastics with 75% level of tomato ketchup contamination		21
	Plastics with 25% level of mayonnaise		21
	Plastics with 50% level of mayonnaise		21
	Plastics with 75% level of mayonnaise		21

**Figure 2:**
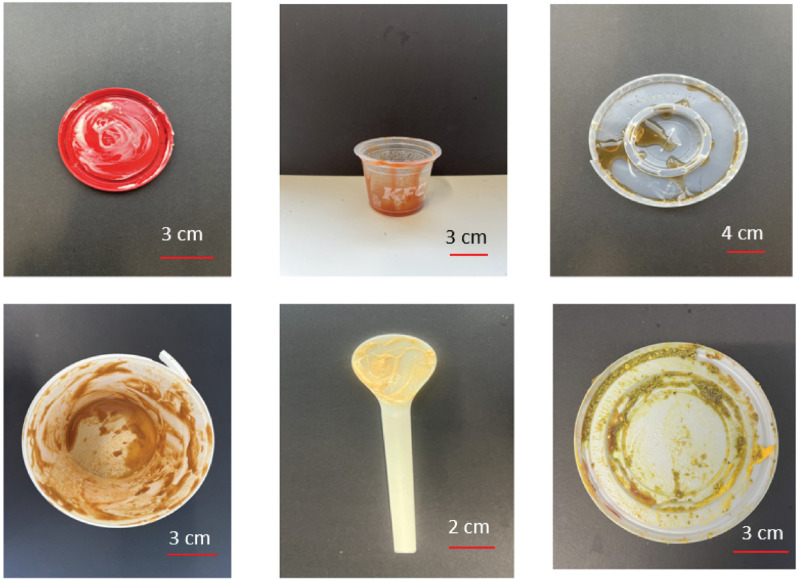
The example of real-world food-contaminated plastic packaging used in the testing dataset.

In the testing dataset, 30 food waste contaminated plastic packaging items were collected from various sources spanning across the city of London including tubs, trays, lids and plastic spoons. These sources were inclusive of both supermarkets, cafes and restaurants, resulting in a variety of packaging types, including take-away boxes, cutlery, lids and more. We selected only the plastic packaging that had a label to show type of packaging in order to verify the model. Figure 2 provides examples of contaminated food packaging in the testing dataset.

The distribution of training, cross-validation and testing datasets was 54%, 36% and 10%. This distribution reflected a strong focus on training the model, supported by a well-balanced validation dataset and a thoughtfully allocated testing set to ensure comprehensive evaluation.

### Imaging methodology

HSI captures a continuous spectrum for each pixel of an object, enabling precise material identification based on unique spectral signatures. Unlike NIR, HSI generates spatial maps that visualise material distribution allowing multi-materials to be detected in a single piece of packaging. Unlike NIR it can detect heterogeneity of contamination, and analyse complex mixtures with unparalleled precision [3,16].

#### Hyperspectral data acquisition and analysis schematic

**Figure 3:**
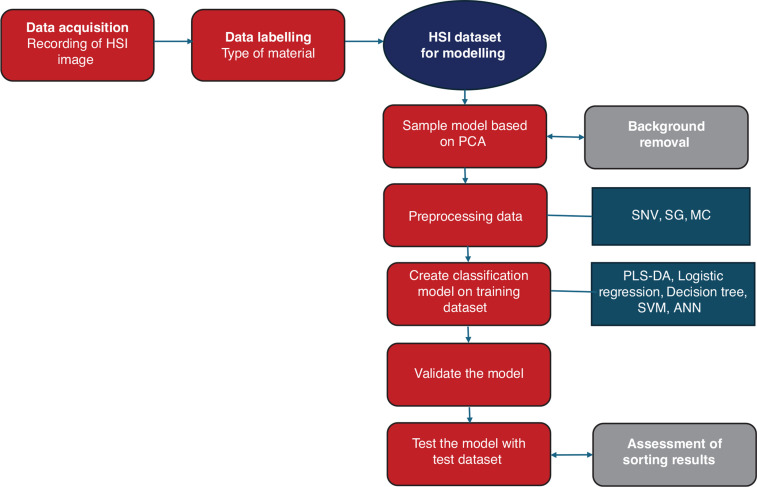
Schematic showing the hyperspectral data acquisition and analysis.

As shown in Fig. 3, hyperspectral images were obtained from line scans of the samples on a conveyor belt passing under a HySpex Baldur S-640i N camera (manufactured by HySpex, Norway). The camera, positioned at a working distance of 1 m with a 16° field of view, covered a spectral range of 950–1730 nm with a spectral resolution of 3.36 nm, resulting in a total of 232 spectral bands. The spatial pixel size of the images was 0.44 mm [17]. The system’s conveyor belt measured 700 mm in length, 215 mm in width and 60 mm in height, with speed of 120 mm/s. The image capture background was the black conveyor belt. A halogen lamp, emitting light across the spectrum from 400 nm to 2500 nm, was employed as the light source. This experimental setup has been described in more detail in our previous work [11,12]. HyspexGround software facilitated the acquisition of the hyperspectral image. Subsequently, the Breeze software package was employed for principal component analysis (PCA) model development, spectrum preprocessing, application of diverse ML algorithms for classification and the production of classification results.

#### Principal component analysis and spectrum preprocessing

PCA was utilised to investigate the relationships between samples and measured variables, with the objective of unveiling patterns within the data. Its primary focus lies in identifying common features rather than distinguishing differences between classes [18]. PCA breaks down data into linear combinations of the original hyperspectral data, known as principal components (PCs). PC1 represents the greatest variability within the dataset, capturing the majority of the information. The subsequent PCs follow in descending order, representing the remaining variance. In our case, PCA was employed to eliminate background pixel and isolate objects (plastics) within the hyperspectral images.

Subsequently, spectral preprocessing was conducted using a combination of methods. This included applying a combination of Savitzky–Golay (SG) first derivative with a second polynomial and a 15-point window, standard normal variate (SNV) and mean centring (MC). This technique was employed to eliminate insignificant baseline signals from the collected data and to rectify scatter data [11].

#### Machine learning classification model

Various ML algorithms, including logistic regression, decision tree (DT) algorithms, support vector machines (SVM), artificial neural networks (ANN) and PLS-DA, were applied to build classification models. The samples in the training dataset were used to develop these models.

##### Logistic regression

Logistic regression is a fundamental supervised learning method widely utilised for classification tasks, particularly in scenarios involving binary outcomes. Through the sigmoid function, it transforms spectral band values to produce probabilities for binary predictions, with coefficients assigned to each band indicating their predictive influence [19,20].

Logistic regression can be extended to address multiclass classification problems through softmax regression. The softmax function normalises the output into a probability distribution across multiple classes, ensuring that the sum of the predicted probabilities for all classes equals unity. This way, the model can provide predictions for each class, and the class with the highest probability is considered as the final prediction [21].

##### Decision tree

A DT is a non-parametric model structured as a tree, where each node contains a decision rule based on input data. This rule directs whether to move to the left or right sub-nodes, while the leaf nodes provide the final output. DTs are applicable to both classification and regression tasks and are particularly valued for their interpretability. One common method for building nodes in a DT is information gain, which uses entropy or the Gini index to measure the amount of information retained by each feature in the input data before making predictions [22].

##### Support vector machine

An SVM was used as a supervised ML algorithm for classification, regression and outlier detection. It was used to identify hyperplanes in the feature space that separate data points belonging to different classes. The hyperplane was positioned to maximise the margin, which is the distance between the hyperplane and the nearest data points of each class. The SVM operates in the original feature space, but kernelised SVMs were also used, these transform data into higher-dimensional spaces through kernel functions. The algorithm requires labelled training data to learn and relies on support vectors, which are crucial points closest to the hyperplane. In a One-versus-One (OvO) approach, binary classifiers are created for each pair of classes. For N classes, this results in C(N, 2) binary classifiers. In our scenario, where we sought to classify seven types of plastics, we used 21 binary classifiers.

##### Artificial neural network

The architecture of an ANN typically comprises three layers: input, hidden and output. The input layer captures spectral information from HSI, where each input to the ANN is a vector representing the spectral signature of each sample. The hidden layer, containing numerous neurons, performs computations on the input data. Hidden layers enable ANNs to learn complex problems and nonlinear relationships. Each neuron in a hidden layer calculates a weighted sum of its inputs, applies an activation function, and produces an output that becomes the input for the next layer. Various activation functions, such as linear, sigmoid, tanh and ReLU, can be employed based on the task.

The input to the ANN was represented by a vector that encapsulates the spectral information, with its length determined by the number of spectral bands or channels in the hyperspectral data. Each element of the vector corresponded to the intensity or reflectance value of the pixel in a specific spectral band. The hidden layer, with 100 neurons, utilised the ReLU activation function to process the hyperspectral data and extract relevant features for classifying the types of plastics [23]. The output layer produced the final classification results, with each neuron corresponding to a different type of plastic, typically using a softmax activation function to provide probabilities for each class.

##### Partial least squares discriminant analysis

PLS-DA, a blend of partial least squares regression (PLS-R) and discriminant analysis (DA), is a supervised ML method for dimensionality reduction and material class prediction. It necessitates an X matrix with calibration spectra and a corresponding Y matrix denoting class identity (types of plastic). In binary cases, Y is a single column; for multiclass scenarios, it is a dummy matrix with 1s and 0s indicating class membership. The model’s output is not strictly binary, requiring a threshold establishment during prediction. Setting thresholds employs various methods, with Bayes’ Theorem being a prevalent choice. Alternatively, a 0.5 cut-off point is often employed for binary classification tasks [24]. In our PLS-DA, the linear equation was modelled with around five latent variables (LVs), enabling graphical visualisation and understanding through LV scores and loadings.

### Classification model performance (model validation)

Model validation is a crucial step in ML, particularly for assessing the performance of classification models. Various metrics are utilised for evaluation, including sensitivity (Equation 1), specificity (Equation 2), precision (Equation 3), F1 score (Equation 4) and accuracy (Equation 5). The formulas for these metrics are based on the following definitions: true positive represents instances where the model correctly predicts the positive class, while true negative indicates instances where the model correctly predicts the negative class. False positive refers to instances where the model incorrectly predicts the positive class, and false negative denotes instances where the model incorrectly predicts the negative class:

(1) $$\text{sensitivity}\;\text{(recall)}\;=\frac{\text{true}\;\text{positive}\;}{\text{true}\;\text{positive}\;+\;\text{false}\;\text{negative}}$$

(2) $$\text{specificity}\;=\text{​}\frac{\text{true}\;\text{negative}\;}{\text{true}\;\text{negative}\;+\;\text{false}\;\text{positive}}$$

(3) $$\text{precision}\;\text{​}=\text{​}\;\frac{\text{true}\;\text{positive}}{\text{true}\;\text{positive}\;+\;\text{false}\;\text{positive}}$$

(4) $$\text{F1}\;\text{score}\;=\frac{\text{true}\;\text{positive}}{\text{true}\;\text{positive}\;+\frac{\text{1}}{\text{2 }}(\text{false}\;\text{positive}\;+\;\text{false}\;\text{negative})}$$

(5) $$\text{accuracy}\;=\frac{\text{true}\;\text{negative}\;\;+\;\text{true}\;\text{positive}}{\text{true}\;\text{negative}\;\;+\;\text{true}\;\text{positive}\;\;+\;\text{false}\;\text{negative}\;\;+\;\text{false}\;\text{positive}}$$

### The evaluation of plastic features in the testing dataset

To measure the impact of plastic features on the performance of classification models, the properties of plastics in the testing dataset, including darkness, level of contamination and size, were evaluated using image processing algorithms to ensure precise evaluation [11].

#### Size

The plastic packaging images in the testing dataset were resized to 10 cm × 15 cm and converted to greyscale. Otsu’s thresholding method was then applied to remove the background and convert the greyscale images to binary format. In this process, pixels with values below the threshold were set to 0, while those above the threshold were set to 255. Following this, the percentages of foreground and background areas were calculated. These percentages were then multiplied by 150 cm^2^ (the total area of the frame) to determine the area occupied by the plastic packaging. The size was classified into three categories: small (<20 cm^2^), medium (20 cm^2^ ≤ area < 80 cm^2^) and large (≥80 cm^2^) [11].

#### Level of contamination

K-means clustering was applied to assess the level of contamination in plastic packaging within the testing dataset. The images were loaded and converted to greyscale, with each pixel represented as a vector based on its greyscale intensity. We selected the number of clusters to be 3. The centroids of each cluster were initialised, and for each pixel in the image, a similarity measure was calculated to determine its proximity to each cluster centroid using a distance metric, such as Euclidean distance. Based on this calculation, the pixel was assigned to the cluster with the closest centroid, forming the initial clusters. Upon convergence, the algorithm produced the final clustering results. At this stage, each pixel was firmly assigned to a specific cluster, and the cluster centroids represented the average greyscale intensities of the pixels within their respective clusters. The number of pixels in each cluster was counted, and their ratios were calculated to determine the percentage of contamination. The level of contamination in the plastic packaging was classified into four categories: low contamination (<25%), medium contamination (25% ≤ contamination <60%), high contamination (≥60%), and indeterminate due to multicoloured packaging or oily contamination [11].

#### Darkness

The images in the testing dataset were loaded and converted into greyscale. Otsu’s threshold theory was applied to separate foreground and background. The average pixel of foreground was calculated to determine the darkness level. The darkness level was classified into three categories: bright (≥157), dark (<157) and transparent [11].

## Results

The results of this study are presented to evaluate the effectiveness of the developed classification models and the impact of plastic attributes, such as size, darkness and contamination levels, on accuracy. The section begins with an analysis of the raw and pre-processed spectra, followed by a comparison of model performances. Finally, the influence of plastic packaging attributes on classification accuracy is demonstrated.

### Average raw absorbance spectrum and pre-processed spectrum

**Figure 4:**
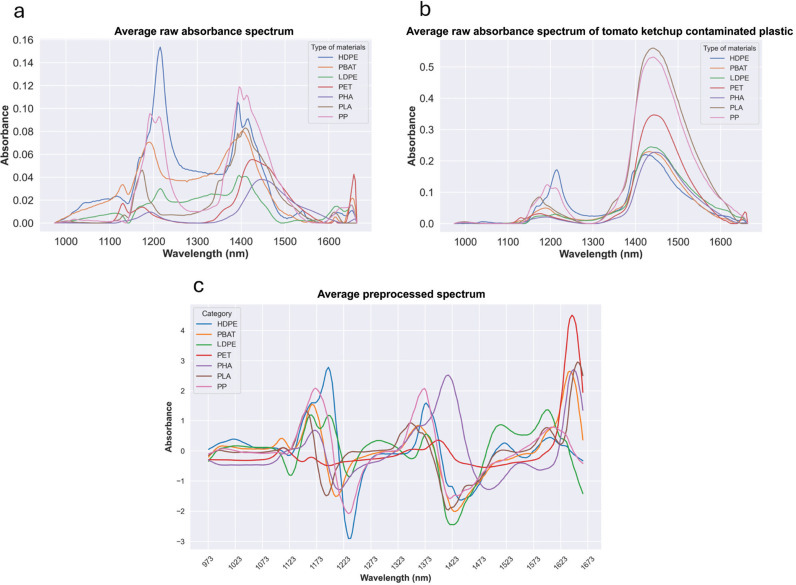
Raw absorbance spectrum of (a) pristine plastics PP, PET, LDPE, HDPE, PLA, PBAT and PHA; (b) the same plastics with 25% of plastic surface contaminated with tomato ketchup; and (c) pre-processed absorbance spectrum of plastics in training dataset (pristine and contaminated with tomato ketchup).

Samples of seven types of plastics including conventional plastic (PP, LDPE, HDPE and PET) and compostable plastics (PLA, PBAT and PHA) were passed underneath the HSI camera by a conveyor belt. The data obtained was used to develop an identification and classification model of plastics with tomato ketchup contamination using ML algorithms. Raw absorbance spectrum of pristine plastic samples and plastic samples with 25% of surface covered with tomato ketchup were shown in Fig. 4a and b, respectively. Raw absorbance of these materials in training dataset was pre-processed using Savitzky–Golay (first derivative, second polynomial and 15 points window) method to identify spectral signatures. The pre-processed absorbance spectra are shown in Fig. 4c.

### Principal component analysis

**Figure 5:**
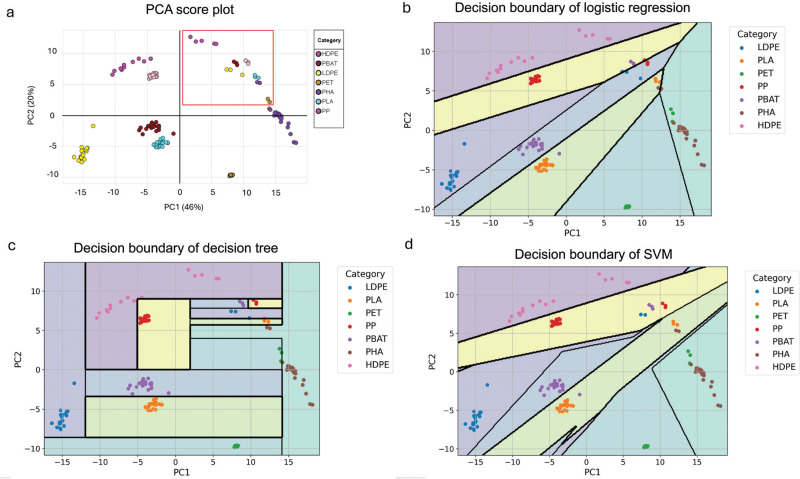
(a) PCA score plot of training dataset, contaminated plastics identified within red box; (b) logistic regression; (c) DT; and (d) SVM for the training dataset.

Following the preprocessing of the absorbance spectrum, a PCA was carried out to achieve dimensional reduction. The spectra of pristine and tomato ketchup contaminated plastics from the training dataset were then utilised to generate a PCA score plot, as depicted in Fig. 5a. The results indicate that a substantial portion of the variance is effectively captured by the first principal component (PC1), which accounts for 46%, and the second principal component (PC2), which contributes 20%. Pristine plastics showed a high level of separability. Specifically, pristine HDPE and PP are situated in the second quadrant, while LDPE, PBAT and PLA are in the third quadrant, and PET and PHA are in the fourth quadrant. Plastics contaminated with tomato ketchup, which are indicated by the red box, are all located in the first quadrant but show some overlap with each other.

### Performance of classification models

#### Performance of classification models on a calibration dataset

The calibration dataset consists of pristine plastics and plastics with the low level of tomato ketchup (25%). We have applied five ML techniques to build classification model on training dataset. Figure 5b–d illustrates the decision boundary of logistic regression, DTs and SVM classification, respectively, providing a visual representation of how these algorithms partition the feature space to classify different plastic samples in training datasets. The decision boundary delineates the regions where each class is predicted, offering insights into the complexity and separability of the dataset. This visualisation aids understanding of the underlying behaviour of the models and their ability to discriminate between different classes of plastics based on the provided features. However, ANN and PLS-DA do not have a straightforward decision boundary. ANN operates through complex transformations of the input data. The decision-making process in an ANN involves a series of interconnected neurons with weighted connections. PLS-DA works by finding linear combinations of features that best separate the classes in the data. Unlike traditional classifiers, PLS-DA does not directly define a decision boundary. Instead, it projects the data into a new space where the classes are maximally separated along LVs. Consequently, it is not as intuitive to visualise the decision boundary in the original feature space.

Table 4 shows the performance of each classification model. For logistic regression, SVM, DTs and ANN, sensitivity, specificity and F1 score all reached 1, resulting in an overall accuracy of 100%. Conversely, other models achieved 100% accuracy. However, PLS-DA exhibited slightly lower accuracy (90.6%) due to its increased sensitivity to outliers, which was particularly noticeable when identifying plastics contaminated with tomato ketchup.

**Table 4.** The performance of various ML algorithms in identifying plastics within the training dataset

ML methods	Polymer	Sensitivity (recall)	Specificity	Precision	F1 score	Overall accuracy
Logistic regression, SVM, DT, ANN	PLA	1.00	1.00	1.00	1.00	100%
	PBAT	1.00	1.00	1.00	1.00	
	PHA	1.00	1.00	1.00	1.00	
	PET	1.00	1.00	1.00	1.00	
	PP	1.00	1.00	1.00	1.00	
	HDPE	1.00	1.00	1.00	1.00	
	LDPE	1.00	1.00	1.00	1.00	
PLS-DA	PLA	0.91	1.00	0.99	0.95	90.6%
	PBAT	1.00	1.00	1.00	1.00	
	PHA	0.87	1.00	1.00	0.93	
	PET	0.87	1.00	1.00	0.93	
	PP	0.87	1.00	1.00	0.93	
	HDPE	1.00	1.00	1.00	1.00	
	LDPE	0.91	1.00	0.99	0.95	

#### Performance of classification models on cross-validation dataset

Before testing the model with real-world contaminated food packaging, we applied these models to classify types of materials in a cross-validation dataset to assess the generalisation of data which included a new type of contamination (mayonnaise). The results are summarised in Table 5.

**Table 5.** The performance of classification models on cross-validation dataset

ML methods	Polymer	Sensitivity (recall)	Specificity	Precision	F1 score	Overall accuracy
Logistic regression	PLA	1.00	1.00	1.00	1.00	95%
	PBAT	1.00	1.00	1.00	1.00	
	PHA	0.67	1.00	0.99	0.80	
	PET	1.00	1.00	1.00	1.00	
	PP	1.00	0.94	0.74	0.85	
	HDPE	1.00	1.00	1.00	1.00	
	LDPE	1.00	1.00	1.00	1.00	
SVM	PLA	1.00	1.00	1.00	1.00	94%
	PBAT	0.93	1.00	0.99	0.96	
	PHA	0.67	1.00	0.99	0.80	
	PET	1.00	0.99	0.94	0.97	
	PP	1.00	0.94	0.74	0.85	
	HDPE	1.00	1.00	1.00	1.00	
	LDPE	1.00	1.00	1.00	1.00	
DT	PLA	1.00	1.00	1.00	1.00	88%
	PBAT	0.89	1.00	0.73	0.80	
	PHA	0.60	1.00	1.00	0.75	
	PET	0.85	1.00	0.60	0.70	
	PP	1.00	0.88	0.57	0.73	
	HDPE	1.00	1.00	1.00	1.00	
	LDPE	1.00	0.98	0.87	0.93	
ANN	PLA	1.00	1.00	1.00	1.00	90%
	PBAT	1.00	1.00	1.00	1.00	
	PHA	0.73	1.00	0.99	0.84	
	PET	1.00	1.00	1.00	1.00	
	PP	1.00	0.89	0.60	0.75	
	HDPE	1.00	1.00	1.00	1.00	
	LDPE	0.60	1.00	1.00	0.75	
PLS-DA	PLA	0.60	0.99	0.90	0.72	75%
	PBAT	0.80	0.92	0.62	0.70	
	PHA	0.47	1.00	0.96	0.63	
	PET	0.80	1.00	1.00	0.89	
	PP	0.80	1.00	0.80	0.80	
	HDPE	1.00	1.00	1.00	1.00	
	LDPE	0.80	0.98	0.84	0.82	

The logistic regression model performed well on datasets, achieving 95% accuracy on the dataset. For PLA, PBAT, PET, HDPE and LDPE it achieved perfect scores of 1 for sensitivity, specificity, precision and F1 score. However, it encountered challenges in accurately detecting PHA due to a new type of contamination and the presence of a thin film. Consequently, instances of PHA were misclassified as PP, resulting in a decrease in sensitivity for PHA to 0.67 and a decrease in specificity for PP to 0.94.

The SVM model achieved 94% accuracy. For PLA, HDPE and LDPE it achieved perfect scores of 1 for sensitivity and specificity. However, like logistic regression, its performance declined when classifying PHA and PBAT. The sensitivity of PHA and PBAT was 0.67 and 0.93, respectively. Misclassifications of PHA (66.7%) and PBAT (6.7%) as PET led to decreases in specificity for PP and PET, resulting in values of 0.94 and 0.99, respectively. Consequently, the precision for PBAT, PHA, PP and PET dropped to 0.99, 0.99, 0.74 and 0.94, respectively, and the F1 scores for PBAT, PHA, PP and PET decreased to 0.96, 0.8, 0.85 and 0.97, respectively.

The DT model achieved 88% accuracy, encountering difficulties in accurately identifying PBAT, PET and PHA. Specifically, the sensitivity for PBAT, PHA and PET was 0.89, 0.6 and 0.85, respectively, while other types of plastics achieved a sensitivity of 1. PBAT was often misclassified as PP (13.3%) and LDPE (6.7%), while PET was misclassified as PP (26.7%). PHA suffered misclassifications as LDPE (6.7%) and PP (33.3%). Regarding specificity, PP and LDPE exhibited lower values compared to other plastic types, with scores of 0.88 and 0.98, respectively. Consequently, the precision for PBAT, PP and PET decreased to 0.73, 0.57 and 0.6, respectively. Additionally, the F1 scores for PBAT, PHA, PP and PET were 0.89, 0.75, 0.97 and 0.85, respectively.

The ANN model demonstrated strong overall performance with an accuracy of 90%. In the cross-validation dataset, it achieved excellent sensitivity, specificity and F1 scores for all types of plastic except for PHA and LDPE, where sensitivity dropped to 0.73 and 0.6, respectively. Furthermore, the model exhibited misclassifications, 40% of LDPE being incorrectly labelled as PP, while 26.7% of PHA samples were misclassified as PP. Additionally, the specificity of PP was low at 0.89. Consequently, the precision for PHA and PP dropped to 0.99 and 0.6 and the F1 scores for PHA, PP and LDPE were computed as 0.84, 0.75 and 0.75, respectively. Overall, while the model achieved impressive accuracy and performance for most plastic types, there are evidently areas for improvement, particularly in accurately distinguishing PHA and LDPE, as well as reducing misclassifications, especially between LDPE and PP.

The performance of PLS-DA fell short compared to other ML algorithms, achieving an overall accuracy of only 75%. Due to the introduction of a new type of contamination (mayonnaise), misclassifications occurred across various plastic types: 6.7% of PBAT, 20% of PET, 13.3% of PLA, 20% of PP and 46.7% of LDPE could not be identified. Additionally, 13.3% of PBAT samples were misclassified as LDPE. Misclassifications were observed between various plastic types as well, with 20.3% of LDPE and 26.7% of PLA incorrectly labelled as PBAT, while 6.7% of PHA samples were misclassified as PLA. Consequently, the sensitivity of PHA was the lowest at 0.47, followed by PBAT, LDPE, PET and PP at 0.8, while PLA had a sensitivity of 0.6. For specificity, all polymers in the cross-validation dataset achieved values greater than 0.9, indicating strong performance in correctly identifying true negatives. However, PLA, PBAT and PP exhibited slightly lower specificity compared to others, with values of 0.99, 0.92 and 0.98, respectively. Additionally, the precision for PLA, PBAT, PHA and PP was 0.9, 0.62, 0.96 and 0.8, respectively, the F1 score for PHA was the lowest at 0.63, followed by PBAT, PLA, LDPE, PET and PP, which achieved scores of 0.7, 0.72, 0.8 and 0.89, respectively.

**Figure 6:**
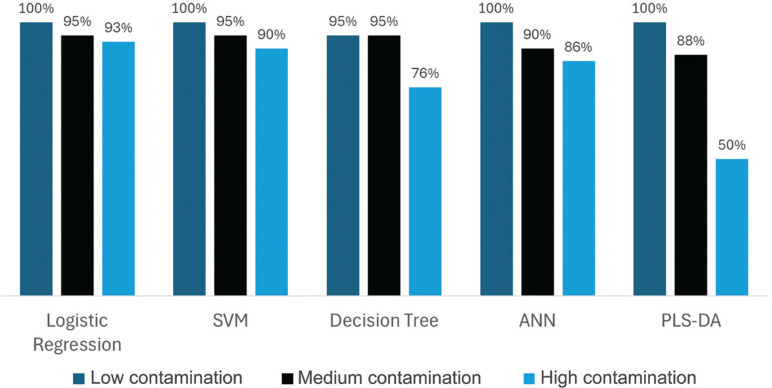
The impact of contamination level on the accuracy of the model.

Figure 6 demonstrates the impact of contamination levels on the accuracy of various classification models. For plastic with a low level of contamination, logistic regression, SVM, ANN and PLS-DA achieved 100% accuracy, while the DT model reached 95% accuracy. As contamination levels increased to a medium level (50%), the accuracy of all models decreased: logistic regression and SVM dropped to 95%, while ANN and PLS-DA fell to 88%. At high contamination levels (75%), the accuracy further declined to 93% for logistic regression, 90% for SVM, 76% for the DT, 86% for ANN and 50% for PLS-DA.

#### Performance of classification models on the testing dataset

The classification models were employed to categorise 30 real-world packaging samples with various types and levels of contamination. The performance of each classification model is shown in Table 6.

**Table 6.** The prediction accuracy of SVM, logistic regression, DT, ANN and PLS-DA on the testing dataset

ML methods	Polymer	Sensitivity (recall)	Specificity	Precision	F1 score	Overall accuracy
SVM	HDPE	1.00	1.00	1.00	1.00	97%
	PET	0.86	1.00	0.98	0.92	
	PHA	1.00	1.00	1.00	1.00	
	PLA	1.00	1.00	1.00	1.00	
	PP	1.00	0.94	0.94	0.97	
	Overall	0.97	0.99	0.99	0.98	
Logistic regression, DT, ANN	HDPE	1.00	1.00	1.00	1.00	93%
	PET	1.00	1.00	1.00	1.00	
	PHA	1.00	1.00	1.00	1.00	
	PLA	1.00	0.96	0.67	0.80	
	PP	0.86	1.00	0.99	0.92	
	Overall	0.81	0.99	0.93	0.94	
PLS-DA	HDPE	1.00	1.00	1.00	1.00	90%
	PET	1.00	1.00	1.00	1.00	
	PHA	1.00	1.00	1.00	1.00	
	PLA	1.00	0.89	0.40	0.57	
	PP	0.79	1.00	0.99	0.88	
	Overall	0.96	0.98	0.87	0.89	

**Figure 7:**
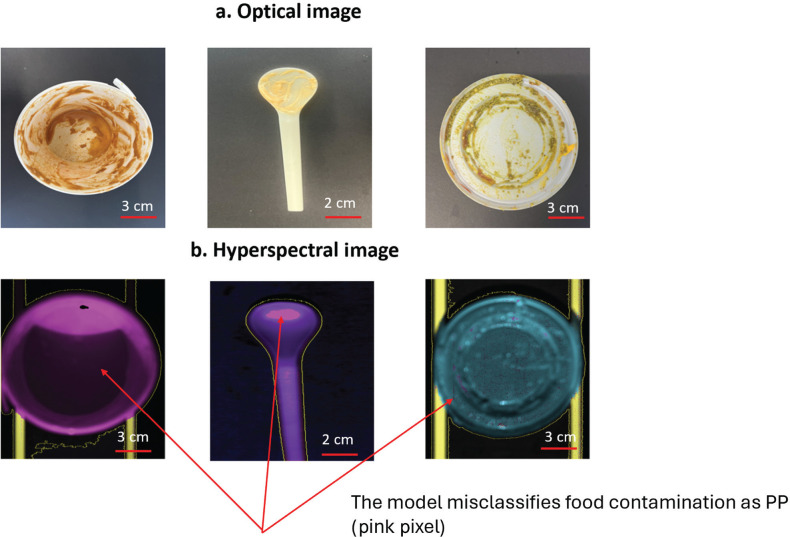
The example of (a) optical Images and (b) hyperspectral images of the testing dataset (PP: pink, PHA, purple, PLA: blue).

From Table 6, it is evident that the overall accuracy of SVM surpasses that of other ML algorithms. However, SVM exhibits lower sensitivity in detecting PET packaging compared to other types of plastic. This is mainly due to the limited reflectance demonstrated by PET, resulting in a weak short-wave infrared (SWIR) signal. Consequently, identifying thin or transparent materials such as PET becomes inherently challenging. Additionally, the model tends to classify contamination as PP, leading to a lower specificity for PP compared to other material types see Fig. 7.

**Figure 8:**
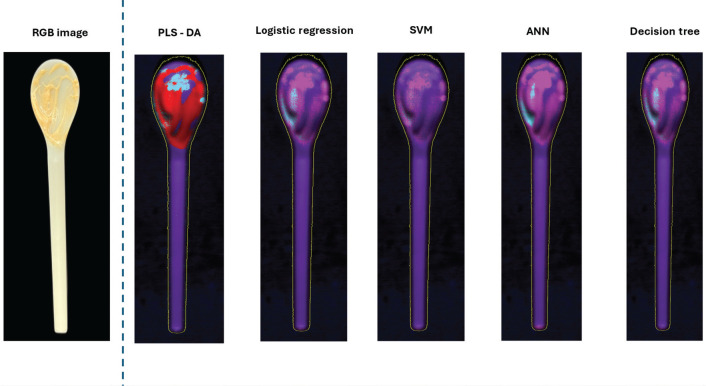
Material classification of PHA spoon with spicy mayonnaise using various ML models (purple pixel: PHA, blue pixel: PLA, pink pixel: PP and red pixel: unidentified pixel).

For logistic regression, DT and ANN, the sensitivity in detecting PP was the lowest at 0.86. PP was misclassified as PLA and LDPE. Thus, the specificity and precision and F1 score of PLA was 0.96, 0.67 and 0.8, respectively, while other types of plastics were 1. The overall accuracy of these models was 93%. The misclassified samples have translucent colour and dark colour. Logistic regression, DT and ANN misclassified a translucent PP lid and a red dark colour Japanese rice bowl as PLA and LDPE, respectively. Some pixels were misclassified by each model, leading to the same final classification outcome. For example, in Fig. 8, we used various classification models to identify the type of food-contaminated spoon. The majority of pixels were classified as PHA. However, for spicy mayo contamination, SVM classified it as PP, while DT, logistic regression and ANN each classified spicy mayo as a combination of PP (pink) and PLA (blue) but in different positions. PLS-DA classified it as a mix of unidentified pixels (red) and PP (pink).

For PLS-DA, the overall accuracy was the lowest at 90%. For PP 21.4% were classified incorrectly as PLA. Therefore, the sensitivity of PP dropped to 0.79 and the specificity of PLA decreased to 0.89. For precision and F1-score, PLA achieved only 0.4 and 0.57, PP achieved 0.99 and 0.88 while others were 1.

Figure 8 shows a sample (spoon) made from PHA (purple) with some contaminated areas (spicy mayonnaise) incorrectly classified as PLA (blue) or PP (pink). This highlights a limitation of PLS-DA in accurately identifying specific materials in contaminated regions compared to other algorithms used in the study.

### Material properties of contaminated plastic packaging in the testing dataset

In this set of experiments, we investigated which properties of contaminated plastic have an impact on the accuracy of selected classification models. Specifically, we measured the size of packaging, the level of contamination and darkness of the packaging.

#### Size

The size of packaging was determined through a surface area estimation algorithm. The average size of plastic packaging in the testing dataset (30 real-world plastic packaging) was 63.94 cm^2^. The number of small, medium and large packaging was 8, 11 and 11, respectively.

**Figure 9:**
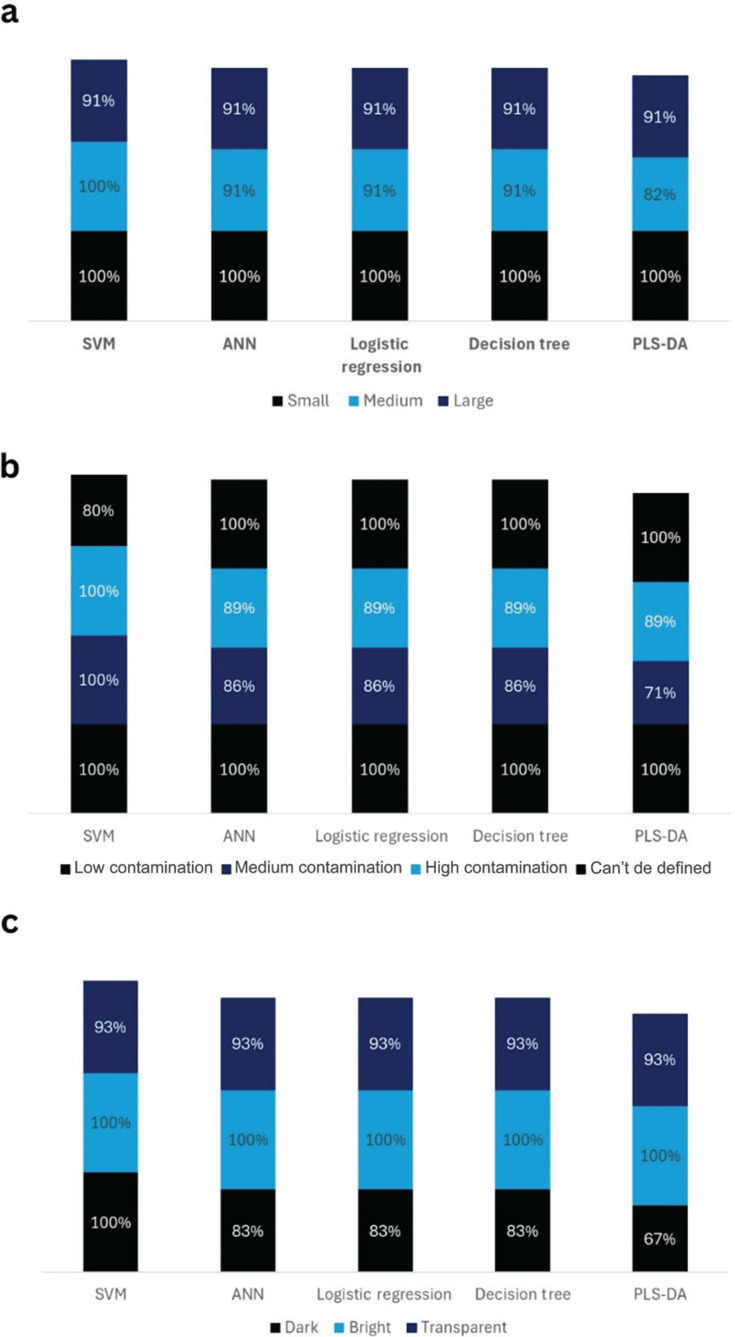
Accuracy of models for identifying plastic samples from the testing data set; (a) small, medium and large plastics; (b) low, medium and high level of contaminated plastic packaging; (c) transparent, bright and dark plastic.

The results are shown in Fig. 9a. For SVM, the system achieved 100% accuracy for small and medium plastic packaging but experienced a drop to 91% accuracy for large plastic packaging. Similarly, for ANN, logistic regression and DT models, the accuracy in detecting small plastic packaging was 100%. For PLS-DA, the accuracy in identifying small plastic packaging was 100%, but it decreased to 82% and 91% when detecting medium and large sizes, respectively. The accuracy of the models dropped when detecting large and medium plastics, as some samples in these categories have opaque colours. However, the accuracy for detecting brightly coloured plastics, regardless of size, is 100%. Thus, sizes larger than 8 cm^2^, which is the size of the smallest plastic packaging, have no impact on the model’s accuracy.

#### Level of contamination

The average level of contamination of real-world plastic packaging was measured at 37%. Figure 9b illustrates the accuracy of the system in identifying types of polymers with several degrees of contamination. In the testing dataset, there were nine plastic packaging samples with a low level of contamination, 10 with medium contamination, and six with a high contamination level. Five pieces of the plastic packaging could not have their contamination level measured due to the presence of labels and transparent oily contamination.

The SVM models performed best, the level of contamination had a low impact on the accuracy of the system. Even with the highest level of contamination reaching 83%, the model still correctly identified the plastic. For ANN, logistic regression and DT models, the accuracy of the model in identifying low-level contaminated plastic was 100%, but it decreased to 86% and 89% when identifying medium and highly contaminated packaging, respectively. Similarly, for PLS-DA, the accuracy of the model in identifying plastic with a low level of contamination was 100%, but it dropped to 71% and 89% when identifying medium and high levels of contamination in plastic packaging, respectively. The PLS-DA model performed better in detecting highly contaminated plastics compared to medium-contaminated plastics, due to the varying levels of darkness in the samples. Most medium contamination samples were dark in colour, while the majority of high contamination samples were bright in colour.

#### Darkness level

The darkness level was identified using the average pixel value of a greyscale image of each sample in the testing dataset. The testing dataset consisted of 15 transparent plastics, six dark coloured plastics and nine brightly coloured plastics. The average darkness level was found to be a greyscale value of 157. The impact of darkness on successful identification is shown in Fig. 9c.

The accuracy of SVM to detect dark coloured and bright coloured plastic was 100% but dropped to 93% when identifying transparent plastic. For ANN, logistic regression and DT, the accuracy of models in identifying dark plastic and transparent was 83% and 93%, respectively, the accuracy increased to 100% when identifying brightly coloured plastics. For PLS-DA, the accuracy to identify dark plastic (67%) was much lower than identifying brightly coloured plastic (100%). However, the accuracy in identifying transparent plastic was 93%.

#### Food contaminant colour

**Figure 10:**
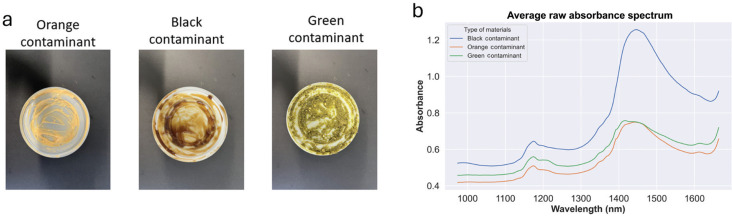
Effect of colour of contamination: (a) PLA lid with orange, black and green contaminant; and (b) the raw absorbance spectrum of PLA packaging with various colours of contaminant (black, orange and green).

The colour of the contaminant exerted a significant impact on the accuracy of the system. This effect is attributed to its influence on the darkness of the material. The interplay between colour and darkness proved to be a crucial factor affecting the model’s performance. Figure 10a displays a PLA lid surface with applied food contamination indicators in black, yellow and green colours.

Black contaminants absorb most of the light, green contaminants absorb less light than black, and orange contaminants absorb very little light, see Fig. 10b. However, our classification models are robust due the use of HSI; it can correctly identify the type of plastic even when the contaminant is black. This result aligns with our previous research [11].

## Discussion

### Classification model performance comparison

The combination of HSI and ML has been applied to identify and classify types of plastics with various types of contamination and contamination level. The samples in this experiment included conventional plastics (PP, PET, LDPE and HDPE) and compostable plastics (PLA, PBAT and PHA). In the training dataset, ML including SVM, DT, logistic regression and ANN achieved 100% accuracy. Even when classifying the plastic with a low level of tomato ketchup, the models still have impressive performance. On the other hand, PLS-DA demonstrated the lowest accuracy among the models, registering a rate of 90.6% in identifying plastic samples within the training dataset.

To enhance the robustness of the model and mitigate overfitting, the utilisation of a cross-validation dataset is crucial. Table 5 explains the accuracy of algorithms on the cross-validation dataset, revealing that logistic regression and SVM exhibit superior performance. These models perform well on the cross-validation dataset, indicating that they can handle new, unseen data effectively including various levels of mayonnaise contamination on the sample surface.

ANN and DT models exhibited accuracy rates of 90% and 88% respectively in identifying contaminated plastic samples within the cross-validation dataset. These models encountered challenges, particularly in misclassifying PHA samples with high mayonnaise contamination.

The obstacles faced by models in detecting contaminated PHA samples are multi-faceted. Firstly, the inherent characteristics of thin and transparent films pose difficulties, given their low absorption in the short-wave infrared (SWIR) range. HSI relies on the absorption of light by molecular vibrations, and when dealing with thin and transparent films, the limited absorption features become a hurdle for the sensor to effectively detect and differentiate materials. Secondly, the presence of thin films introduces scattering effects, causing alterations in the direction of incident light. This scattering effect can introduce variability in the measured spectra, creating challenges in maintaining the consistency required for reliable classification. Thirdly, contamination on the surface of PHA induces shifts in the absorbance spectrum, further complicating the classification process. The introduction of contaminants alters the characteristic molecular vibrations, making it challenging for the models to accurately identify and categorise the material. PLS-DA may face challenges when dealing with intricate relationships within the data, especially in scenarios where the underlying patterns are highly complex. Moreover, the dataset is small, so other machine learnings performed better than PLS-DA.

### Influence of material properties on the performance of the models

Our focus extended to three material properties: size, level of contamination and darkness. Analysis revealed no discernible correlation between the size of the material (particularly when exceeding 8 cm^2^) and the accuracy of the model. Surprisingly, the level of contamination demonstrated minimal influence on the system’s accuracy. Darkness showed significant impact on the accuracy of the system. Opaque plastic is more difficult to classify due to the high light absorbance of the SWIR region. Transparent plastic is also difficult to identify due to the scattering of light.

Black contaminants absorb most of the light, green contaminants absorb less light than black, and orange contaminants absorb very little light, see Fig. 10b. However, our classification models are robust; they can correctly identify the type of plastic even when the contaminant is black. This result aligns with our previous research [11].

Our SVM model for identifying polymer contamination performed comparably to the PLS-DA model developed by Bonifazi, both achieving a sensitivity of 0.97. However, our model was applied to packaging with a higher level of contamination [10]. Additionally, Cucuzza’s Hierarchical PLS-DA model demonstrated impressive accuracy, reaching up to 1.0. These findings highlight that the integration of HSI with ML significantly enhances the recycling rate by accurately identifying polymer contamination [25]. Kraśniewski applied various ML techniques to identify 11 types of polymers, finding that PET had the lowest accuracy due to its transparency, which aligns with our results [26]. Importantly, our SVM model developed here enhances the performance of our previous PLS-DA model [12]. Even with highly contaminated packaging, the SVM model can identify polymers with 100% accuracy. In contrast, our previous PLS-DA model could only accurately identify pristine plastics, with accuracy dropping to 75% for highly contaminated plastics.

### Application of hyperspectral imaging in anaerobic digestion, in-vessel composting and recycling plant for detecting food-contaminated compostable plastics

In AD and IVC, the first step involves sorting the waste. Pre-consumer waste, often referred to as source-separated, includes a wide range of organic materials and other contaminants. The primary task is to remove all packaging and separate organic matter from non-organic materials such as metals, minerals, dirt and various unexpected objects. This ensures that only appropriate organic materials are processed further, improving efficiency and output quality [27].

Depackaging and separation are carried out using machines called depackagers. The reject stream from these machines consists of packaging materials, including contaminated plastics, cardboard, glass and metal. After the separation process, IVC primarily relies on manual sorting combined with visual inspection to identify compostable plastics, which is labour-intensive and costly [28]. Contaminated plastics that cannot be composted are sent to landfill or incineration. Similarly, in AD, all contaminated plastics are directed to landfill or incineration [3].

The integration of HSI with ML methods can enhance the system by reducing the landfill and incineration of plastics and increasing recycling and composting rates. With a detection system in place, compostable plastics can be reintroduced into the system, and recyclable plastics can be detected and sent to recycling plants.

If recycling plants employed this detection system, a high percentage of food-contaminated compostable plastics could be identified and redirected to composting facilities for proper processing. Additionally, the system could help identify the 17% of recyclable plastics that are rendered non-recyclable due to food contamination [29]. Implementing this system would require an automatic separation system to act on identification and characterisation provided by the HSI system. These already exist in modern waste recycling facilities. They are less common in AD and IVCs. Investment in these facilities would have to be driven by a return on investment which is incentivised by the lower number of plastics being sent to landfill and incineration. The next steps in development would be to trial this system in a commercial AD or IVC setting where the effect of the speed of the conveyor belt on accuracy, and other real-time variables could be tested.

## Conclusion

In this study, we demonstrated that the combination of hyperspectral imaging and ML algorithms can effectively identify different types of plastic packaging, even when highly contaminated with food waste. Five ML algorithms including SVM, logistic regression, DT, ANN and PLS-DA were applied to classify real-world contaminated plastic packaging. Among these, SVM achieved the highest accuracy at 97%, followed by logistic regression, ANN and DT, achieving 93%. PLS-DA showed the lowest accuracy at 90%. However, the model faced challenges in identifying PET due to its transparency.

Furthermore, the influence of material attributes, including size, contamination level and darkness, was evaluated. Darkness significantly impacted the system’s accuracy, as opaque plastics were more challenging to classify due to more light absorption in the SWIR region. In contrast, size and contamination level had a lesser effect on the models’ accuracy.

This technology has the potential to be implemented in real waste management facilities, addressing current challenges and significantly improving the efficiency of waste sorting systems. As a result, recycling and composting rates could increase, contributing to the creation of a circular economy for plastic waste.

## Data Availability

The datasets generated during and/or analysed during the current study are available from the corresponding author on reasonable request.
